# Evaluation of Immunization Route in Induction of Vaccine-Mediated Anti-Gonococcal Immune Responses in a Murine Model of Ascending Infection

**DOI:** 10.1093/infdis/jiaf445

**Published:** 2025-09-16

**Authors:** Kathryn A Matthias, Kristie L Connolly, Ann E Jerse, Ogan K Kumova, Andrew N Macintyre, Mary C Gray, Keena S Thomas, Alison K Criss, Ryszard A Zielke, Lixin Li, Aleksandra E Sikora, Fidel Ramirez-Bencomo, Angela Thistlethwaite, Jeremy P Derrick, Wei-En Lu, Margaret C Bash

**Affiliations:** Center for Biologics Evaluation and Research, U.S. Food and Drug Administration, Silver Spring, Maryland, USA; Department of Microbiology and Immunology, Uniformed Services University of the Health Sciences, Bethesda, Maryland, USA; Department of Microbiology and Immunology, Uniformed Services University of the Health Sciences, Bethesda, Maryland, USA; Center for Biologics Evaluation and Research, U.S. Food and Drug Administration, Silver Spring, Maryland, USA; Duke Human Vaccine Institute and Department of Medicine, Duke University School of Medicine, Durham, North Carolina, USA; Department of Microbiology, Immunology, and Cancer Biology, University of Virginia School of Medicine, Charlottesville, Virginia, USA; Department of Microbiology, Immunology, and Cancer Biology, University of Virginia School of Medicine, Charlottesville, Virginia, USA; Department of Microbiology, Immunology, and Cancer Biology, University of Virginia School of Medicine, Charlottesville, Virginia, USA; Department of Pharmaceutical Sciences, College of Pharmacy, Oregon State University, Corvallis, Oregon, USA; Department of Pharmaceutical Sciences, College of Pharmacy, Oregon State University, Corvallis, Oregon, USA; Department of Pharmaceutical Sciences, College of Pharmacy, Oregon State University, Corvallis, Oregon, USA; Vaccine and Gene Therapy Institute, Oregon Health and Science University, Beaverton, Oregon, USA; School of Biological Sciences, Lydia Becker Institute of Immunology and Inflammation, The University of Manchester, Manchester, United Kingdom; School of Biological Sciences, Lydia Becker Institute of Immunology and Inflammation, The University of Manchester, Manchester, United Kingdom; School of Biological Sciences, Lydia Becker Institute of Immunology and Inflammation, The University of Manchester, Manchester, United Kingdom; Center for Biologics Evaluation and Research, U.S. Food and Drug Administration, Silver Spring, Maryland, USA; Center for Biologics Evaluation and Research, U.S. Food and Drug Administration, Silver Spring, Maryland, USA

**Keywords:** *Neisseria gonorrhoeae*, gonorrhea, outer membrane vesicles (OMVs), vaccine, ascending infection

## Abstract

**Background:**

Identification of immune correlates in murine gonorrhea models has been hampered by study-dependent differences in vaccine antigens and administration routes. We previously showed that detergent-detoxified outer membrane vesicles (dOMVs) isolated from a PorA-, PorB-, and RmpM-deficient meningococcal strain (ΔABR) elicit antibodies that cross-react with *Neisseria gonorrhoeae* and enhance gonococcal clearance in a mouse model of lower reproductive tract infection. In this study, we investigated whether (1) ΔABR dOMVs can protect mice from ascending gonococcal infection and (2) vaccination route influences immune responses.

**Methods:**

Mice were vaccinated subcutaneously (SC) or intraperitoneally (IP) and then vaginally inoculated with gonococci. Bioburden of mice was measured and assessed relative to ΔABR dOMV-induced cellular and humoral immune responses.

**Results:**

Subcutaneous and intraperitoneal vaccination accelerated gonococcal clearance from the lower and upper reproductive tract at similar rates. Probing of gonococcal protein microarrays with immune sera from the 2 groups identified multiple vaccine targets that were commonly immunogenic. Despite comparable clearance patterns in vaccinated mice, differences in immune induction were observed that were dependent on administration route. SC immunized mice demonstrated a neutrophil influx that correlated with decreased vaginal bioburden; higher serum bactericidal activity against nonsialylated gonococci was also noted. In contrast, IP immunization induced higher serum and vaginal IgA levels, serum bactericidal activity against sialylated gonococci, and antigonococcal opsonophagocytic killing activity of neutrophils.

**Conclusions:**

This work demonstrates that ΔABR dOMVs protect against ascending gonococcal infection and that cellular and functional antibody responses to the same candidate vaccine may vary depending on immunization route.

## Teaser

Murine gonococcal infection models provide valuable information regarding host immune responses but are complicated by study design differences. In this report, we show that meningococcal vesicles protect against ascending gonococcal infection, with immune responses that are dependent on vaccination route.

With an estimated global incidence of 82.4 million and the identification of isolates resistant to all known antibiotic classes, *Neisseria gonorrhoeae* (*Ng*) constitutes an urgent threat to public health [1]. Although most mucosal infections are uncomplicated, *Ng* can ascend in both sexes, causing inflammatory infections that may result in infertility if left untreated. Identification of correlates of protection is complicated by the host restriction of *Ng* for humans and the lack of immunity to repeat infections. The controlled human infection model can aid in assessment of anti-*Ng* immune responses [2] but is specific to male urethritis due to sequelae associated with ascending infection in women [3]. Estradiol-treated mouse models permit study of *Ng* infections in the female lower reproductive tract (LRT) and can include transgenic mice expressing human complement regulators [4] and colonization receptors [4, 5]. While these models do not require iron supplementation to establish *Ng* infection [6], administration of exogenous human transferrin (hTF) is essential for infection of the upper reproductive tract (URT), where availability of soluble iron is restricted. The hTF-supplemented mouse model does not require transcervical inoculation, but promotes ascending infection following vaginal inoculation, with high numbers of *Ng* recovered from the endometrium and oviducts as late as 10 days postinfection [7].

We previously reported that detergent-detoxified outer membrane vesicles (dOMVs) isolated from a meningococcal mutant deleted for the major outer membrane proteins (OMPs) PorA, PorB, and RmpM (ΔABR) enhance *Ng* clearance in a murine LRT model [8]. These studies were conducted using intraperitoneal (IP) immunizations, which are common for murine immunogenicity studies but do not recapitulate human vaccination. Other groups have tested IP vaccination in the mouse infection model [9, 10], though some have employed more translational routes (ie, subcutaneous [SC], intramuscular, intravaginal, and intranasal) [7, 9, 11–14]. As use of the mouse model becomes more common in the search for gonococcal correlates of protection, a systematic evaluation of the influence of vaccination route on immune readouts is needed.

In this report, we used an hTF-supplemented mouse model to determine whether (1) ΔABR dOMVs could protect against ascending gonococcal infection and (2) vaccination route influences immune responses. Selection of IP and SC vaccination routes allowed us to bridge our results back to previous studies [8] and to evaluate whether potential immune correlates were specific to nontranslational immunization models.

## METHODS

### Bacterial Growth Conditions

ΔABR was incubated on Brain Heart Infusion agar supplemented with 5% heat-inactivated equine serum or in Tryptic Soy Broth medium, both with 50 μg/mL kanamycin. *Ng* strain F62 was cultured on supplemented GC agar. Commensal flora and F62 isolated from vaginal swabs were grown, respectively, on Heart Infusion Agar and GC-VNCTS agar (GC agar with vancomycin, colistin, nystatin, and trimethoprim supplement and 100 μg/mL streptomycin). All media were purchased from Difco.

### Vaccine Production

dOMVs were extracted from ΔABR and detoxified using sodium deoxycholate buffers [15]. Following ultracentrifugation, dOMVs were suspended in PBS, and protein and lipooligosaccharide (LOS) levels estimated as previously described [15]. LOS levels were comparable with those previously reported (<10 endotoxin units per μg dOMV) [15].

### Ethics Statement

Animal experiments were conducted at the Uniformed Services University or the Food and Drug Administration according to guidelines established by the Association for the Assessment and Accreditation of Laboratory Animal Care, using protocols approved by the respective institutions’ Institutional Animal Care and Use Committees.

### Dose-Ranging and Immunogenicity Studies

For dose-ranging studies, female 4-week-old BALB/cAnNCr mice (Charles River, *n* = 5 mice per group) were administered 3 SC doses of 6.25, 12.5, or 25 μg ΔABR dOMVs prepared 1:1 with alhydrogel (alum) adjuvant (InvivoGen) at 3-week intervals; 3 IP doses of 12.5 μg ΔABR dOMVs with alhydrogel (*n* = 5) or alhydrogel given SC alone (*n* = 10) were also administered as controls. Immunogenicity studies were similarly conducted, except that 12.5 μg of ΔABR dOMVs with alhydrogel were administered SC (ΔABR-SC) or IP (ΔABR-IP) (*n* = 10 mice per group). Controls were given alum SC or IP (*n* = 5 mice per route). Blood and vaginal lavage samples were collected at study termination.

### Ascending Infection Model

The ascending mouse model of *Ng* infection was performed as described [7] with immunizations conducted using the immunogenicity study protocol (*n* = 20 mice per group). Briefly, 3 weeks post-3rd immunization, mice were implanted SC with 17β-estradiol pellets (Innovative Research of America). Two days later, in-stage mice (*n* = 18 for ΔABR-IP and alum, *n* = 19 for ΔABR-SC) were injected IP with hTF (8 mg, Sigma) and vaginally inoculated 4 hours later with 10^5^ CFUs of *Ng* strain F62; daily hTF injections were given to infected mice until study termination. Vaginal swabs for quantitative *Ng* culture and polymorphonuclear leukocyte (PMN) smears were collected up to +7 days postchallenge. At day 7, blood and vaginal lavage samples were collected, mice were humanely sacrificed, and the URTs were removed for quantitative culture. See Supplementary Materials for detailed methods.

### Cell Phenotyping

Single cell suspensions of iliac lymph nodes or spleens (2.0 × 10^6^) were labeled with LIVE/DEAD™ Fixable Aqua Dead Cell Stain (Thermo Fisher) and surface monoclonal antibody (mAb) markers described in Supplementary Table 1. Cells were permeabilized, labeled with cytokine-specific mAbs (Supplementary Table 1), and fixed in 1% paraformaldehyde for processing on an LSRFortessa™ X-20 flow cytometer (BD Biosciences). Results were analyzed using FlowJo software (Tree Star). For some experiments, cells were stimulated ex vivo with 1 μg/mL of ΔABR membrane preparations [16] for 6 hours prior to labeling.

### ELISA

Three hundred eighty-four-well microtiter plates were coated with 80 ng/well of F62 OMVs [17]. Total IgG, IgG1, IgG2a, IgG2b, IgG3, IgA, and IgM antibodies were measured from serum and vaginal lavage samples as endpoint titers largely as described [18] using a BioMek automated liquid handler.

### Opsonophagocytic Killing Activity Assay

Opsonophagocytic killing activity (OPKA) assays were performed as described [19] using PMNs from healthy human donors following protocols approved by the University of Virginia Institutional Review Board for Health Sciences Research. PMNs (2 × 10^5^) were incubated with 5% C6-depleted normal human serum (Complement Technology) and *Ng* strain FA1090 preopsonized with heat-inactivated test serum. Aliquots were plated onto GC agar and incubated overnight. Percent bacterial survival was calculated as: [(CFUs with PMNs)/(CFUs without PMNs)] × 100.

### Human Complement Serum Bactericidal Activity Assays

Human complement serum bactericidal activity (hSBA) was tested against F62 with nonsialylated (NS) or sialylated (S) LOS. The NS-hSBA assay was performed as described [19], by incubation of F62 with serially diluted heat-inactivated test sera and 4% pooled IgG/IgM-depleted normal human complement serum (Pel-Freez). The S-hSBA was performed as described [20] by culturing F62 with 50 μg/mL of CMP-NANA (Nacalai), then incubating bacteria with test sera and 10% IgG/IgM-depleted complement. For the NS-hSBA assay, results were reported as the highest reciprocal dilution at which ≥50% bacteriolysis was observed relative to controls (bacteria incubated with complement). Titers were determined by the same formula for the S-hSBA and used to calculate the hSBA Index: (hSBA titer with active complement)/(hSBA titer with heat-inactivated complement).

### Immunoblots

Membrane preparations [16] of *Neisseria* strains were fractionated by sodium dodecyl sulfate polyacrylamide gel electrophoresis, transferred to iBlot2 polyvinylidene fluoride membranes (Invitrogen), and probed with pooled serum or vaginal lavages. An HRP-conjugated goat anti-mouse IgG antibody (Bio-Rad) was applied, and the blots were developed with Pierce ECL Western Blotting Substrate (Thermo Fisher).

### Protein Microarray

Gonococcal microarray slides were printed by Arrayjet Ltd using proteins produced recombinantly in-house [21, 22]. Slides were blocked, probed with pooled mouse sera, and DyLight® 650-conjugated goat antimouse IgG (Abcam) applied. The slides were scanned using an InnoScan 710 (Innopsys) system, and data quantified using Mapix—Microarray image acquisition and analysis software (v9.1.0, Innopsys). Microarray spot intensities were calculated using automatic background subtraction and were recorded in quintuplicate. Data represent the arithmetic mean subtracted for buffer-only controls.

### Statistical Analysis

Applied statistical tests are defined in the corresponding figure legends. All data were graphed and analyzed using GraphPad Prism v7.03 software.

## RESULTS

### Dose-Ranging Study

We first conducted dose-ranging studies to assess comparability of anti-ΔABR dOMV seroresponses when administered SC versus IP. *Ng*-specific antibody levels in sera collected at study termination (3-weeks post-3rd vaccination) demonstrated similar phenotypes for mice vaccinated SC with 6.25 μg ΔABR dOMVs versus those given a 12.5 μg IP dose, though mean IgG2a levels in the SC group were comparatively lower, and IgG1 and IgG3 levels were not significantly higher than alum controls (Supplementary Figure 1*A*–*E*). IgG1:IgG2a ratios were >2 for all vaccinated mice, suggestive of Th2 skewing, with the greatest mean difference observed between mice given 12.5 μg ΔABR dOMVs SC and IP (Supplementary Figure 1*F*). Mice given an SC dose of 6.25 μg versus 12.5 μg ΔABR dOMVs also trended toward a lower IgG1:IgG2a ratio, though the difference was not significant (Supplementary Figure 1*F*).

### Immunogenicity Study

**Figure 1.**
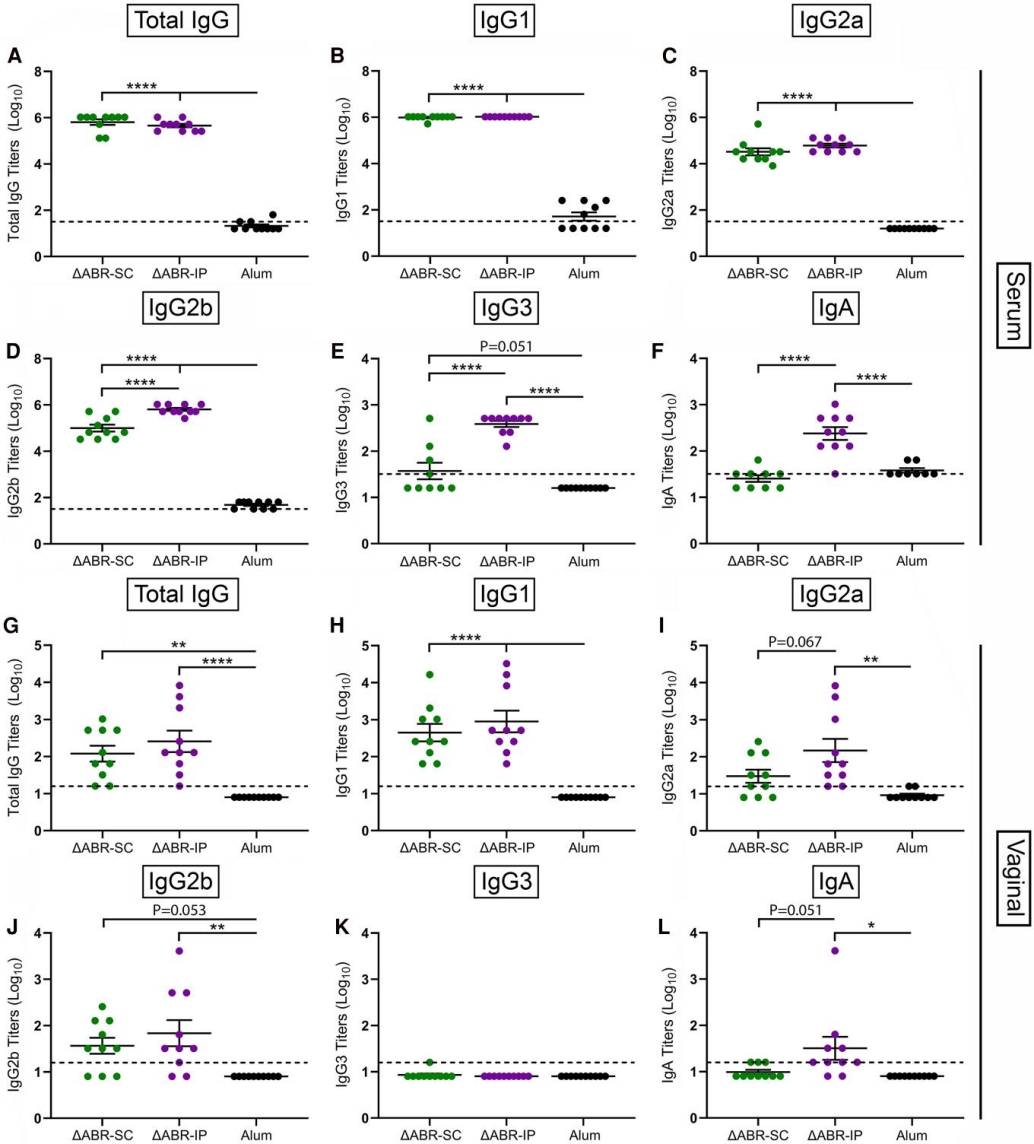
F62-specific (*A*–*F*) serum and (*G*–*L*) vaginal antibody levels present in mice 3-weeks post-3rd immunization. Alum group represents the combined titers of control mice immunized subcutaneously and intraperitoneally. *P < .05, **P < .01, and ****P < .0001 by 1-way ANOVA with Tukey's multiple comparison test. Hashed lines indicate the assay limit of detection (LOD). Samples with nonmeasurable titers were imputed to a value equivalent to half the LOD.

To maximize the likelihood of observing divergent host responses, we selected the 12.5 μg SC and IP doses for further study. We immunized mice (*n* = 10 per group) 3 times at 3-week intervals to establish baseline responses in the absence of *Ng* infection; 5 mice each were also immunized SC or IP with alum alone as negative controls. Robust *Ng*-specific IgG, IgG1, IgG2a, and IgG2b serum antibody titers were detected 3-weeks post-3rd vaccination (Figure 1*A*–*D*). The ΔABR-IP group was also characterized by elevated mean IgG3 and IgA titers (Figure 1*E*—and  *F*). Similar trends were observed in vaginal lavages collected at the same timepoint, though anti-*Ng* IgG2a and IgG2b levels in the ΔABR-SC group were not significantly higher than the alum group, possibly due to variability in lavage collection and mucosal secretions among mice; *Ng*-specific IgG3 was also only detected in a single mouse (Figure 1*G*–*L*).

We next tested the antisera for functional activity. When assessed in OPKA assays, sera from the ΔABR-IP, but not the ΔABR-SC, group significantly enhanced PMN-mediated *Ng* killing (Supplementary Figure 2A). SBA was also detected in sera from vaccinated groups, though the group demonstrating higher seropositivity was dependent on the LOS sialylation state (*ΔABR-SC for non-sialylated and ΔABR-IP for sialylated Ng*, Supplementary Figure 2*B* and *C*). Regression analyses conducted with ELISA, OPKA, and SBA results revealed no correlation among the datasets (data not shown).

### *Ng* Challenge Study

**Figure 2.**
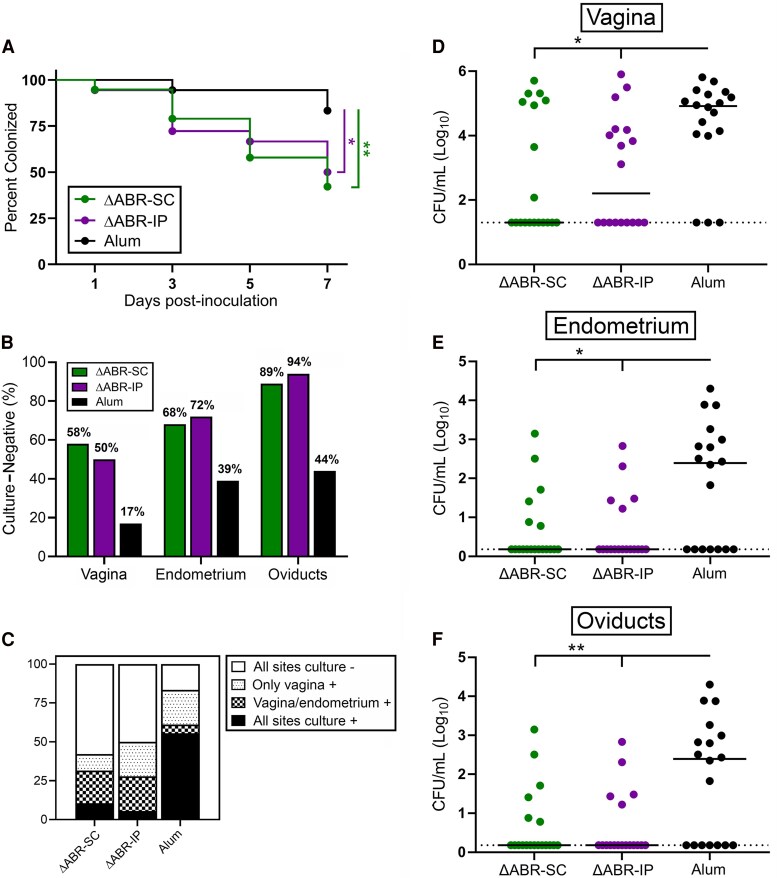
ΔABR detergent-detoxified outer membrane vesicles enhance protection against gonococcal infection. *A*, Percentage of ΔABR-SC, ΔABR-IP, and alum-immunized mice that were culture-positive in the lower reproductive tract over the time course. *P < .05 and **P < .01 by the log-rank (Mantel–Cox) test. *B*, Percentage of mice that were culture-negative in the vagina, endometrium, and oviducts at day 7. *C*, Percentage of mice that were culture-negative or culture-positive at one or more anatomical sites at day 7. *D–F*, Mean CFU/mL recovered from the (*D*) lower and (*E–F*) upper reproductive tract +7 d postchallenge. Hashed lines represent the lower technical limit of CFU quantification. *P < .05 and **P < .01 by Kruskal–Wallis with Dunn's multiple comparison test.

Using the same vaccination schedule, we immunized 20 mice SC or IP with 12.5 μg ΔABR dOMVs or with alum alone (IP). Three weeks post-3rd immunization, mice were challenged vaginally with *Ng* strain F62; swabs were collected on days +1, +3, +5, and +7 postchallenge to enumerate CFUs and PMN infiltrates. At day 7, the study was terminated, and the endometrium and oviducts cultured. A significantly greater proportion of ΔABR-SC and ΔABR-IP mice cleared LRT infection versus alum controls +7 days postchallenge (Figure 2*A*); a greater proportion of vaccinated mice were also culture-negative for *Ng* in the endometrium and oviducts (Figure 2*B* and *C*). Accordingly, the average number of CFUs at all 3 anatomical sites was lower at day 7 for vaccinated mice relative to controls (Figure 2*D*–*F*), and an AUC analysis showed significantly lower vaginal bioburden for mice administered ΔABR dOMVs throughout the infection period (Supplementary Figure 3).

### Evaluation of Protective Immune Responses

**Figure 3.**
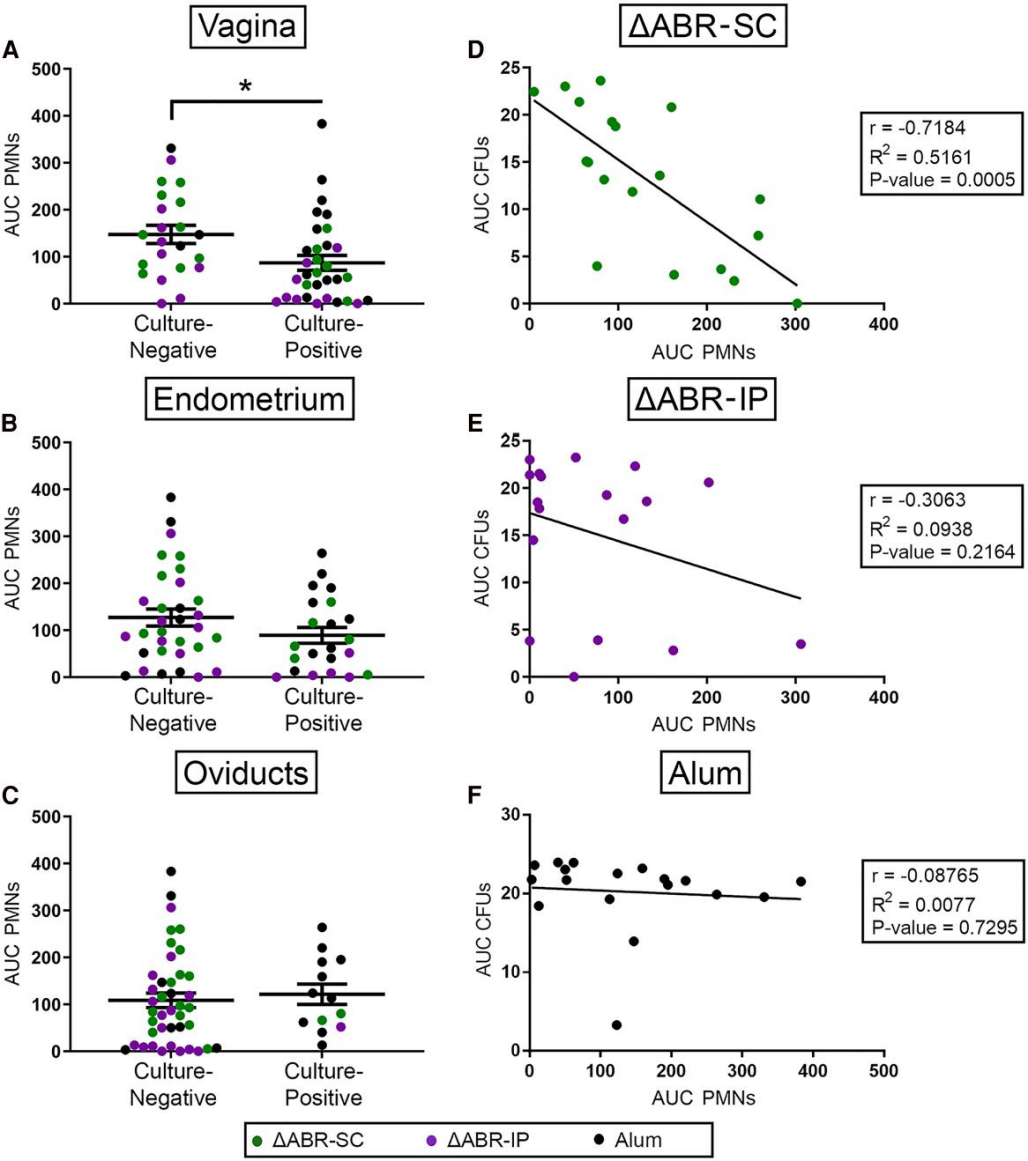
Polymorphonuclear leukocyte (PMN) recruitment is associated with gonococcal clearance from the lower reproductive tract. *A–C*, AUC analysis of PMNs recovered from vaginal swabs at day +1, +3, +5, and +7 postbacterial challenge. The AUC for PMN infiltrates of each mouse was grouped according to whether mice were culture-negative or culture-positive in the (*A*) vagina, (*B*) endometrium, and (*C*) oviducts at day 7. *P < .05 by 2-tailed unpaired Student *t* test. *D*–*F*, Regression analysis (Pearson correlation) of the number of CFUs plotted against the PMNs recovered from vaginal swabs over the infection period. Data were grouped according to whether mice were vaccinated (*D*) subcutaneously or (*E*) intraperitoneally, or were administered (*F*) alum alone.

To identify potential mechanisms of protection, we first assessed cellular responses in mice following *Ng* challenge. Vaginal infection was characterized by PMN influx as early as +1 day postchallenge, though there was no correlation between the number of PMN infiltrates and clearance among immunized groups (data not shown). When categorized by infectious state at study termination, a significant difference in PMN influx was observed for mice that had cleared the LRT independent of vaccination status and route (Figure 3*A*); no significant difference was detected for mice that were culture-negative in the endometrium or oviducts (Figure 3*B* and *C*). A significant inverse correlation between the number of vaginal PMNs and CFUs recovered throughout the infection was found for ΔABR-SC (Figure 3*D*), but not ΔABR-IP (Figure 3*E*) or alum-immunized (Figure 3*F*), mice.

**Figure 4.**
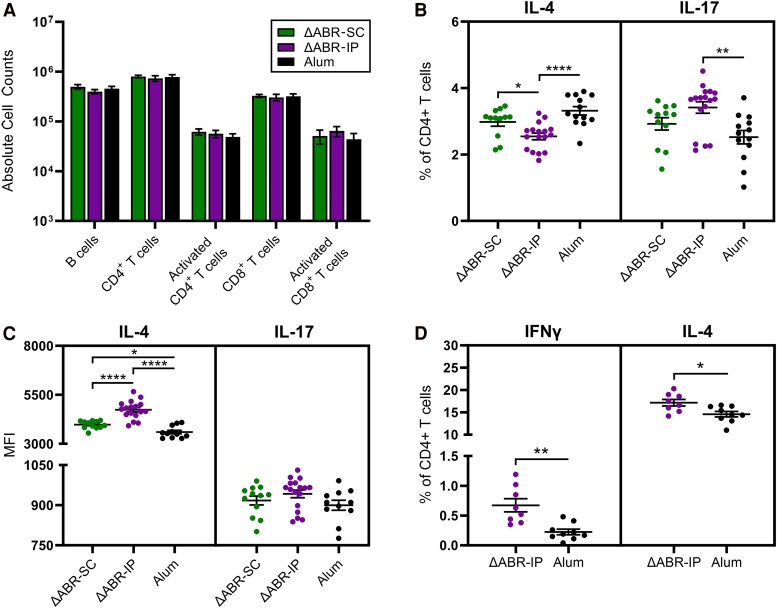
Intraperitoneal vaccination alters T helper cell responses of gonococcal-infected mice. *A*, Absolute number of (activated) B and T cells in iliac lymph nodes as measured by flow cytometry. *B*, Percentage of IL-4+ (Th2) and IL-17+ (Th17) CD4+ T cells in iliac lymph nodes of infected mice. *C*, Mean fluorescence intensity (MFI) of IL-4- and IL-17-expressing CD4+ T cells, as gated on positively-expressing populations. *D*, Percentage of Th1 and Th2 cells in splenocytes of mice stimulated ex vivo with ΔABR membrane preparations. For *B* and *C*, *P < .05, **P < .01, and ****P < .0001 by 1-way ANOVA with Tukey's multiple comparison test. For *D*, *P < .05 and **P < .01 by 2-tailed unpaired Student *t* test.

Using *Ng* infection as a natural stimulant, we next tested iliac lymph nodes and spleens collected at study termination for production of local and systemic cellular responses. Despite similar numbers of activated CD69+ B (CD19+) and T (CD3+CD4+/CD3+CD8+) cells in the lymph nodes (Figure 4*A*), ΔABR-IP-immunized mice contained lower and higher proportions of IL-4+ (Th2) and IL-17+ (Th17) CD4+ T cells, respectively, compared with alum controls (Figure 4*B*). Increased Th2 populations were observed in ΔABR-SC versus ΔABR-IP mice, though no significant differences in Th2 or Th17 populations relative to controls were noted (Figure 4*B*). Robust IFNγ+ (Th1) CD4+ T-cell populations were not detected in any group.

Analysis of the mean fluorescence intensity (MFI) of CD4+ T cells demonstrated production of significantly higher levels of IL-4 on a per-cell basis for both vaccinated groups relative to controls, and for the ΔABR-IP group compared with ΔABR-SC mice (Figure 4*C*). A trend toward increased IL-17 levels was observed for vaccinated mice but was not significant (Figure 4*C*). No differences in T helper cell populations were observed in the splenocytes of immunized mice or in local/systemic CD8+ T cells post-*Ng* infection (data not shown). When stimulated ex vivo with ΔABR membrane preparations, representative ΔABR-IP samples showed increased proportions of Th1 and Th2 cells versus alum controls, highlighting the capacity of vaccinated mice to develop antigen-specific T cell responses (Figure 4*D*).

**Figure 5.**
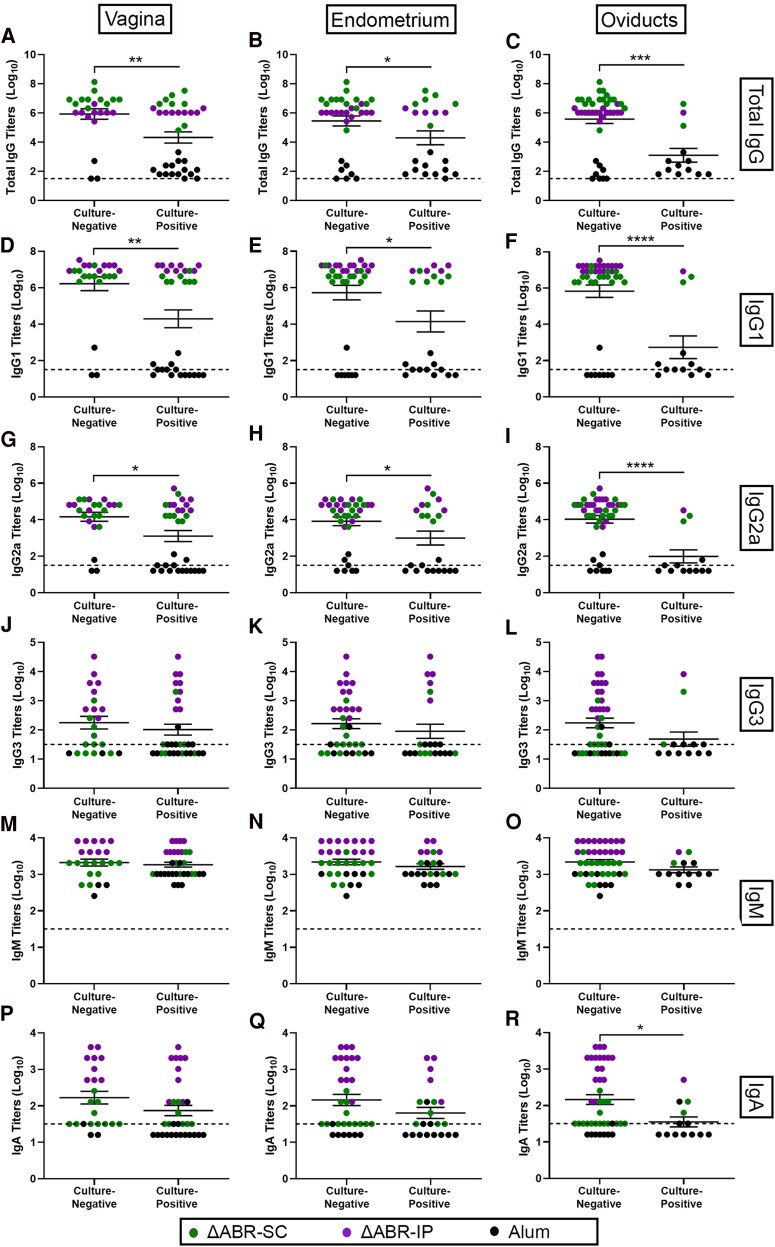
F62-specific serum (*A*–*C*) IgG, (*D*–*F*) IgG1, (*G*–*I*) IgG2a, (*J*–*L*) IgG3, (*M*–*O*) IgM, and (*P*–*R*) IgA antibody levels present in mice +7 d postbacterial challenge. Titers are grouped according to mice that were culture-negative or culture-positive in the (*A*, *D*, *G*, *J*, *M*, *P*) vagina, (*B*, *E*, *H*, *K*, *N*, *Q*) endometrium, and (*C*, *F*, *I*, *L*, *O*, *R*) oviducts at day 7. *P < .05, **P < .01, ***P < .001, and ****P < .0001 by 2-tailed unpaired Student *t* test.

Levels of serum antibodies detected in each group at study termination were comparable with those observed post-3rd immunization in the immunogenicity study, except that the titers of anti-F62 IgM, which we tested in lieu of IgG2b as a marker of early humoral responses, were significantly higher in the ΔABR-IP group (Supplementary Figure 4). To determine if serum antibodies contributed to *Ng* clearance, we grouped the titers of individual mice according to infectious state at the study endpoint. IgG, IgG1, and IgG2a titers were significantly higher in mice that were culture-negative in the vagina, endometrium, and oviducts (Figure 5*A*–*I*). No significant differences were observed in IgG3 or IgM titers (Figure 5*J*–*O*), and elevated IgA titers were only noted in mice that were culture-negative in the oviducts (Figure 5*P*–*R*). Regression analyses revealed that significant differences were heavily weighted by the higher antibody levels present in vaccinated versus unvaccinated mice, and that no correlation existed between antibody titers and bioburden (data not shown), suggesting limited utility of an antibody threshold as a predictor of gonococcal clearance.

**Figure 6.**
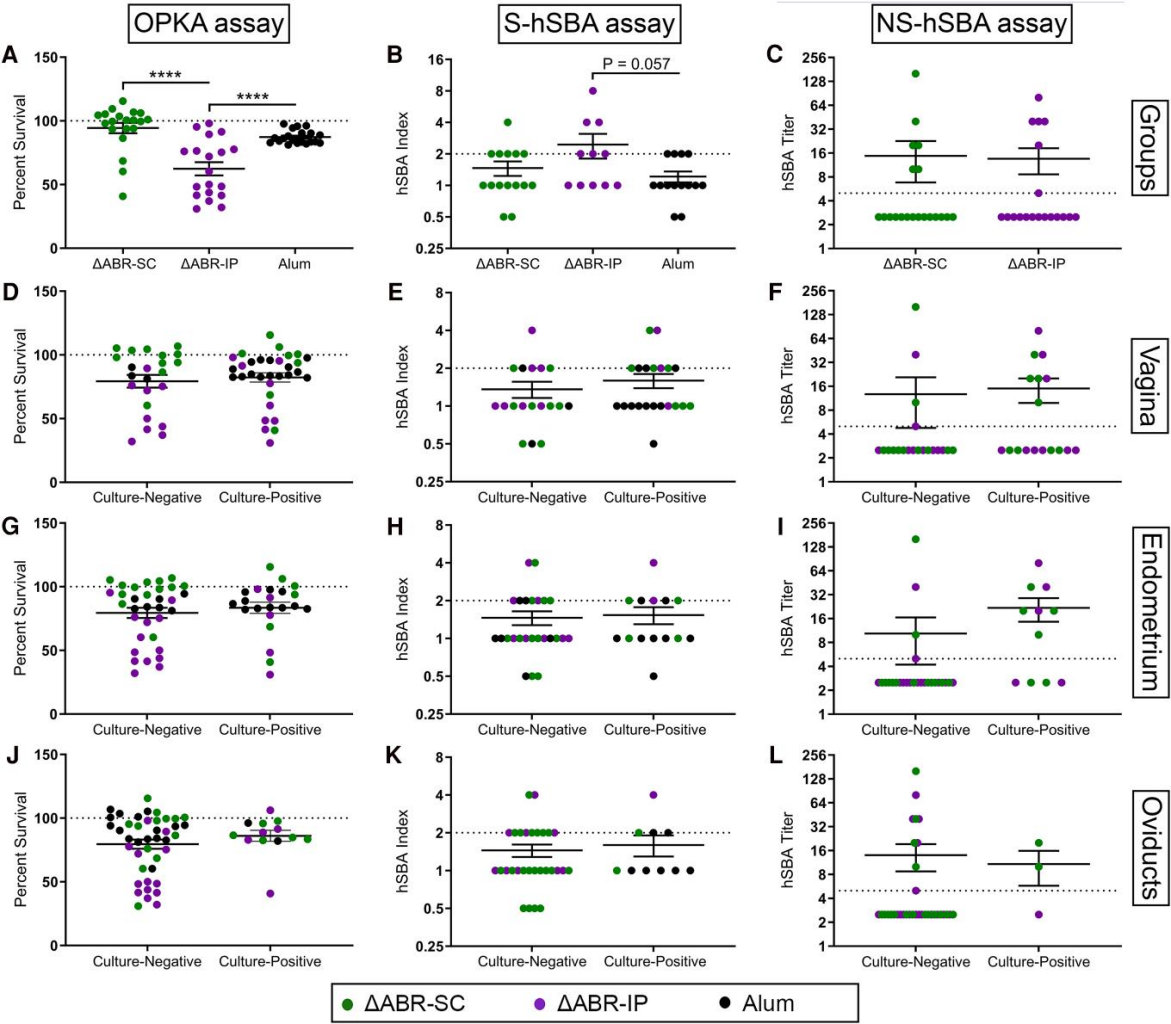
Functional antibody responses postgonococcal infection. *A*, Opsonophagocytic killing activity (OPKA) of serum antibodies of mice +7 d postbacterial challenge. ****P < .0001 by 1-way ANOVA with Holm–Šidák test. *B* and *C*, Serum bactericidal activity (SBA) of antibodies as measured against F62 with (*B*) sialylated and (*C*) nonsialylated lipooligosaccharide. Samples with results less than the limit of detection (LOD) were imputed to a value equivalent to half the LOD. *D*–*L*, Compiled data from *A* to *C* as grouped according to lower and upper reproductive tract infectious state. Samples from mice that were vaccinated but not inoculated with gonococci are excluded. Data were analyzed using (*B*) 1-way ANOVA with Tukey's multiple comparison test and (*C–L*) 2-tailed unpaired Student *t* test. Hashed lines for OPKA and SBA analyses represent no killing and the lower threshold of killing, respectively.

Consistent with the immunogenicity study, antibodies from ΔABR-IP mice demonstrated enhanced OPKA relative to those from ΔABR-SC and alum-immunized mice (Figure 6*A*). Likewise, a trend toward increased S-hSBA seropositivity was observed for the ΔABR-IP group relative to controls (Figure 6*B*), though the difference was not statistically significant. In contrast, NS-hSBA seropositivity, which was highest for the ΔABR-SC group post-3rd immunization, did not differ from that of the ΔABR-IP group postinfection due to an increase in ΔABR-IP mouse antibody titers (Figure 6*C*). No significant differences in OPKA or SBA were observed when combined antibody responses were grouped according to infectious state and anatomical location (Figure 6*D*–*L*), though significantly higher NS-hSBA titers were observed for ΔABR-IP mice that remained culture-positive in the endometrium versus culture-negative mice (*P* = .029 by 2-tailed unpaired Student *t* test, data not shown). No correlation was found among CFUs, OPKA, and SBA in individual mice (data not shown).

### Identification of Immunogenic Antigens

To identify the antigen specificity of anti-ΔABR dOMV antibodies, we probed gonococcal membrane preparations with pooled antisera obtained postchallenge from the immunization groups. Prominent IgG and IgA responses against a ∼72 kDa protein previously identified as the type IV pilin secretin PilQ [8] were observed, with stronger responses noted in the vaccinated groups relative to alum controls (Supplementary Figure 5). Weaker serum and vaginal IgG responses were also observed against a ∼28 kDa antigen and multiple antigens of ∼43–95 kDa in vaccinated mice (Supplementary Figures 5 and 6). IgG and IgA antibodies specific for proteins (∼36–43 kDa) corresponding to the PorB expressed by each of the *Ng* strains (Supplementary Figure 7) were detected in all immunized mice, but only at highly concentrated antibody dilutions (Supplementary Figure 5).

**Figure 7.**
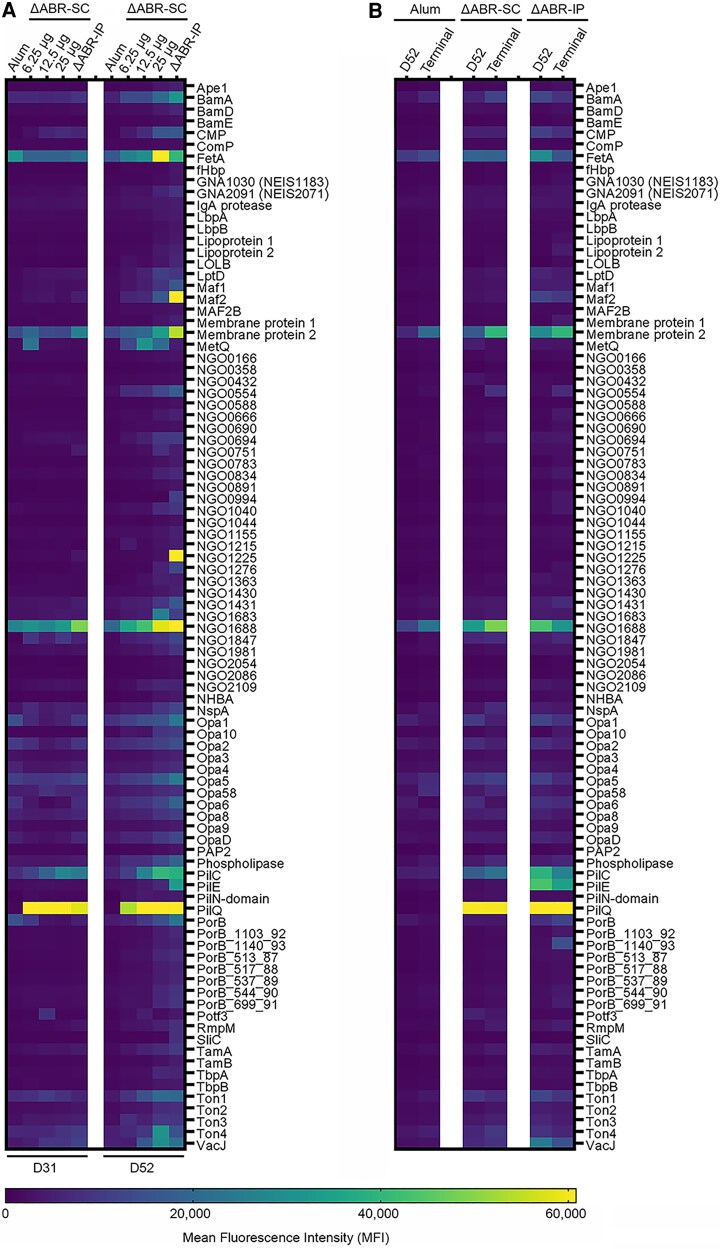
Identification of immunogenic antigens in ΔABR detoxified outer membrane vesicles (dOMVs). *A*, Pooled sera from mice isolated in the dose-response study 10 d post-2nd (D31, left panel) and post-3rd (D52, right panel) immunization were used to probe protein microarrays. *B*, Microarrays probed with sera from mice in the gonococcal challenge study isolated 10 d post-3rd vaccination (D52) or +7 d postchallenge (terminal). Left, middle, and right panels represent arrays probed with pooled sera from alum-, ΔABR-SC-, and ΔABR-IP-immunized mice.

We next probed protein microarrays of 91 unique *Ng* antigens with pooled mouse sera collected during the dose-ranging and infection studies. The results confirmed vaccination with dosages as low as 6.25 μg ΔABR dOMVs elicited strong antibody responses to PilQ, irrespective of immunization route, and weaker antibody responses to the structural pilin biogenesis protein PilC and the phospholipid transporter VacJ (MlaA) (Figure 7*A*). In contrast, some vaccine-dependent antibody responses were route-specific (ΔABR-SC: L-methionine binding lipoprotein MetQ; ΔABR-IP: pilin protein PilE and peptidyl-prolyl cis–trans isomerase NGO1225 [MIP]) (Figure 7*A* and *B*).

Antibodies specific to certain antigens were also observed in all serum samples, including those obtained from uninfected alum-immunized mice (Figure 7*A* and *B*). Higher ΔABR dOMV dosage or number of dOMV immunizations led to increased production of antibodies against these antigens. Strong responses were observed postvaccination to the surface lipoprotein assembly modulator NGO1688 (OmpU), the iron-regulated transporter FetA, and membrane protein 2 (NGO0648). Weaker responses were observed to multiple opacity proteins, the BAM complex transmembrane protein BamA, the Ton-dependent zinc piracy receptor Ton1 (TdfH), and the transferrin-binding protein Ton4 (TbpB). Low levels of PorB-specific antibodies were also present in the sera of each uninfected immunization group (Figure 7*A*), suggestive of antibodies that were cross-reactive for commensal *Neisseria* PorB or other similar antigens.

## DISCUSSION

In this study, we demonstrated that meningococcal ΔABR dOMVs protect against *Ng* vaginal and ascending infection in mice. Protection was independent of IP or SC vaccination route, despite observation of route-specific immune responses. Most notably, a strong inverse correlation between PMNs and vaginal bioburden was observed for the ΔABR-SC group, but not for ΔABR-IP or alum-immunized mice. Similarly, Zhu et al. [23] showed that ΔABR dOMVs, when given IP, and the 4CMenB vaccine, when given SC, enhanced *Ng* clearance from the murine LRT, but vaginal bioburden of the 4CMenB group alone correlated inversely with PMNs.

These data suggest that SC vaccination may enhance epigenetic changes in progenitor/myeloid cells that favor recruitment of hyperresponsive PMNs upon *Ng* infection, a process known as innate immune “training” [24]. Studies with a modified vaccinia virus support this model, as multiple SC, but not intradermal, vaccinations result in rapid and robust engagement of activated PMNs [25, 26]. However, OPKA of ΔABR-SC antisera was significantly lower than that of ΔABR-IP antisera when tested against human PMNs ex vivo. Human PMNs differ from those of mice in production of key factors (eg, defensins and human CEACAM receptors) [27, 28], which may contribute to different mechanisms of *Ng* killing. When wild-type and CEABAC mice expressing humanized PMNs were tested in the LRT infection model, neutropenia was associated with increased percentages of *Ng*-infected mice; the difference was only significant for the CEABAC mice, which were also characterized by a ∼10-fold higher bioburden [5]. These data suggest that humanized and mouse PMNs control murine infection via different mechanisms, though the relevance of route-dependent differences in PMN recruitment and functionality remains unclear.

In addition to OPKA, vaccination route also influenced SBA. While NS-hSBA seropositivity was elevated for the ΔABR-SC versus the ΔABR-IP group in immunogenicity studies, similar seroresponses were noted following *Ng* infection, a result of increased ΔABR-IP antibodies. One possible explanation is that ΔABR-IP mice elicited antibodies against a different repertoire of antigens in the immunogenicity and challenge studies, leading to an overall rise in titers; we were unable to test this theory directly in microarrays due to low immunogenicity study serum volumes. Alternatively, SC and IP vaccination may have favored elicitation of antibodies to different antigens, SC for LOS and IP for proteins. This hypothesis is supported by S-hSBA testing, which shows (1) a consistently low hSBA Index for the ΔABR-SC group, as expected if LOS sialylation blocked anti-LOS antibodies [29, 30] and (2) higher hSBA Index results (>2) for ΔABR-IP mice postinfection, consistent with protein-specific antibody boosting. We detected no anti-LOS antibodies in immunoblots probed with pooled antisera, indicating that the antibodies, if present, were of low concentration. Antibodies against multiple gonococcal OMPs were also detected by microarray independent of vaccination route, calling this theory into question.

Similarities in antibody recognition profiles and clearance rates of ΔABR-SC and ΔABR-IP mice is intriguing considering observation of different route-dependent immune phenotypes. These results suggest that either (1) none of the immune responses identified play a role in mediating *Ng* clearance in mice, (2) *Ng* clearance is mediated via different route-dependent mechanisms, or (3) *Ng* clearance can be mediated through multiple mechanisms. The study by Zhu et al. [23] favors the latter explanation, as mice vaccinated IP with ΔABR dOMV and SC with 4CMenB both enhanced *Ng* clearance, though at different rates and with different immune readouts. For the ΔABR dOMV group, specifically, induction of IgG2a antibodies was associated with decreased bioburden [23]. Our study showed high levels of serum IgG2a antibodies in ΔABR-IP, as well as ΔABR-SC, mice. Vaginal IgG2a antibodies were significantly elevated in ΔABR-IP mice relative to alum controls, a phenotype shown by Liu et al. [12] to be associated with protection of mice from repeated *Ng* infection. The same study suggested that skewing from Th17 to Th1 responses protects mice from *Ng* in an IFNγ- and B cell-dependent mechanism [12].

In our study and others [23], splenocytes from vaccinated mice produced IFNγ and IL-4 when stimulated with *Neisseria* antigens, demonstrating development of potentially protective mixed Th1/Th2 responses. Additional studies will be required to examine the mechanisms by which PMNs and antibodies may contribute to Th1/Th2-mediated protection from gonorrhea.

## Supplementary Material

jiaf445_Supplementary_Data

## Supplementary Data

Supplementary materials are available at *The Journal of Infectious Diseases* online (http://jid.oxfordjournals.org/). Supplementary materials consist of data provided by the author that are published to benefit the reader. The posted materials are not copyedited. The contents of all supplementary data are the sole responsibility of the authors. Questions or messages regarding errors should be addressed to the author.
