# Supplementary material for: Evaluation of Immunization Route in Induction of Vaccine-Mediated Anti-Gonococcal Immune Responses in a Murine Model of Ascending Infection
Source: J Infect Dis. 2025 Sep 16;232(5):e765–77. doi: 10.1093/infdis/jiaf445 (PMC12614976; doi:10.1093/infdis/jiaf445)
Supplement: jiaf445_Supplementary_Data [file jiaf445_supplementary_data.docx]

**SUPPLEMENTARY METHODS**

**Dose-Ranging and Immunogenicity Studies**

Mouse dose-ranging and immunogenicity studies were conducted as described in the primary Methods section. For collection of vaginal lavages, 100 μL of PBS was instilled into the vagina using a blunt-ended pipet tip; the same volume of PBS was pipetted into the vagina three times to maximize acquisition of mucosal secretions. To obtain blood samples, mice were administered xylazine and ketamine intraperitoneally (IP) at study termination, and cardiac puncture was employed. Blood was permitted to clot at room temperature for ≥30 minutes, at which time samples were centrifuged at 2500 x g for 5 minutes. Sera were collected and stored in conjunction with vaginal lavages at -80°C until use.

**Ascending Infection Model**

Four-week-old female BALB/cAnNCr mice (*n*=20, Charles River) were immunized subcutaneously (SC) or IP three times at 3-week intervals with 12.5 μg of ΔABR dOMVs prepared 1:1 with Alhydrogel adjuvant (InvivoGen) diluted in PBS. Alternatively, mice were administered Alhydogel in PBS alone (alum control). Three weeks after the final immunization (day -2), mice in the anestrus or the diestrus stage of the reproductive cycle were implanted SC with a 6.5 mg 21-day slow-release 17β-estradiol pellet (Innovative Research of America) and administered streptomycin sulfate (2.4 mg), vancomycin hydrochloride (0.4 mg), and trimethoprim sulfate (0.4 g/L) as described [1] to suppress the overgrowth of commensal flora under the influence of estrogen. In brief, streptomycin/vancomycin were given as a single IP injection at day -2 and two daily injections at days -1, 0, and +1 relative to gonococcal challenge. Trimethoprim was added to the drinking water from days -2 to +7 relative to gonococcal challenge; streptomycin (5 g/L) was also added to water during the infection period (days 0 through +7).

Two days following initiation of estradiol treatment (day 0), in-stage mice (*n*=18 for ΔABR-IP and alum control groups, *n*=19 for ΔABR-SC group) were administered IP injections of 8 mg of holo-transferrin (hTF, Sigma). Four hours later, mice were vaginally inoculated with 10^5^ CFUs of gonococcal strain F62. Single daily hTF injections were administered throughout the infection period (days +1 through +7 post-challenge), and vaginal swab samples were collected on days +1, +3, +5, and +7 for quantitative *Ng* culture and to prepare stained smears to assess the influx of polymorphonuclear leukocytes (PMNs). Bacteria from swabs were suspended in PBS and plated on GC-VCNTS agar for enumeration, where the limit of detection was equivalent to 20 CFU/mL. PMNs were reported as a percentage relative to the total cell count.

On day 7 post-challenge, venous blood and vaginal lavage samples were collected for antibody measurements. Mice were humanely sacrificed, and their upper reproductive tracts removed after clamping a hemostat above the cervix. The uterine body and horns were sliced longitudinally, rinsed with sterile PBS, and the endometrial tissue was scraped off with a sterile scalpel for suspension in PBS (total volume = 1 mL). GC broth was used to generate serial dilutions of the scrapings, which were cultured on GC-VCNTS agar for enumeration. For oviducts, tissues were minced with a scalpel and rinsed with 100 μL PBS before plating on GC-VCNTS agar. The limit of detection was 1.5 CFU/mL for endometrial washes and 1 CFU/100 μL for oviduct cultures.

REFERENCES

1. Jerse AE, Wu H, Packiam M, Vonck RA, Begum AA, Garvin LE. Estradiol-treated female mice as surrogate hosts for *Neisseria gonorrhoeae* genital tract infections. *Front Microbiol* **2011**; 2:107.

**SUPPLEMENTARY DATA**

**Supplementary Table 1**. List of BioLegend monoclonal antibodies used for cell phenotyping experiments.

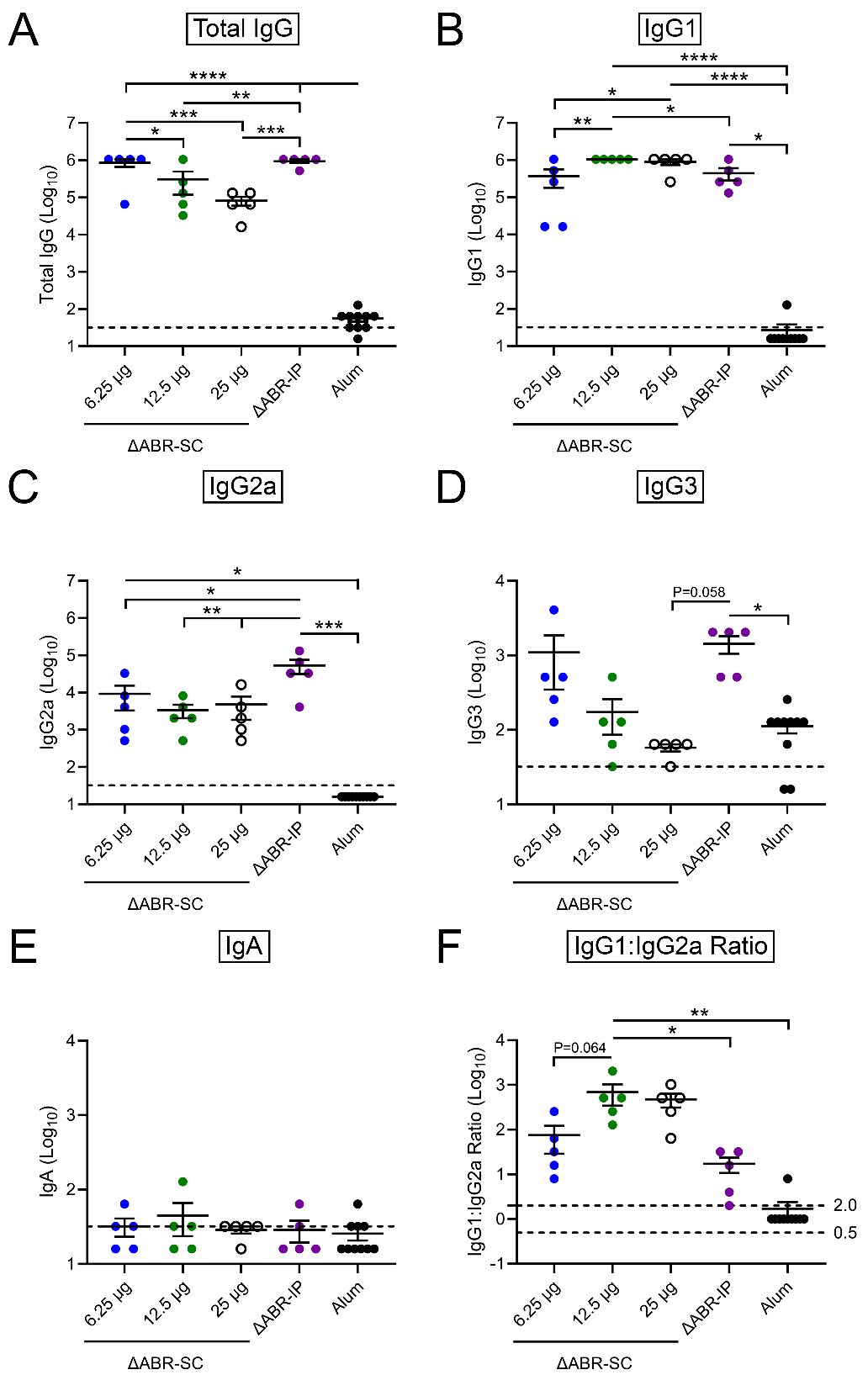


**Supplementary Figure 1.** *A-E*, F62-specific serum antibody levels measured by ELISA 3-weeks post-3^rd^ immunization. Samples with nonmeasurable titers were imputed to a value equivalent to half the limit of detection (LOD). *F*, IgG1:IgG2a ratio as calculated using data from *B*-*C*. *P˂0.05, **P˂0.01, ***P˂0.001, and ****P˂0.0001 by 1-way ANOVA with Tukey’s multiple comparison test. Hashed lines indicate the assay LOD for *A-E* and the upper and lower bounds defining a mixed Th1/Th2 response for *F*, where values ˂0.5 and ˃2 represent Th1- and Th2-skewed responses, respectively.


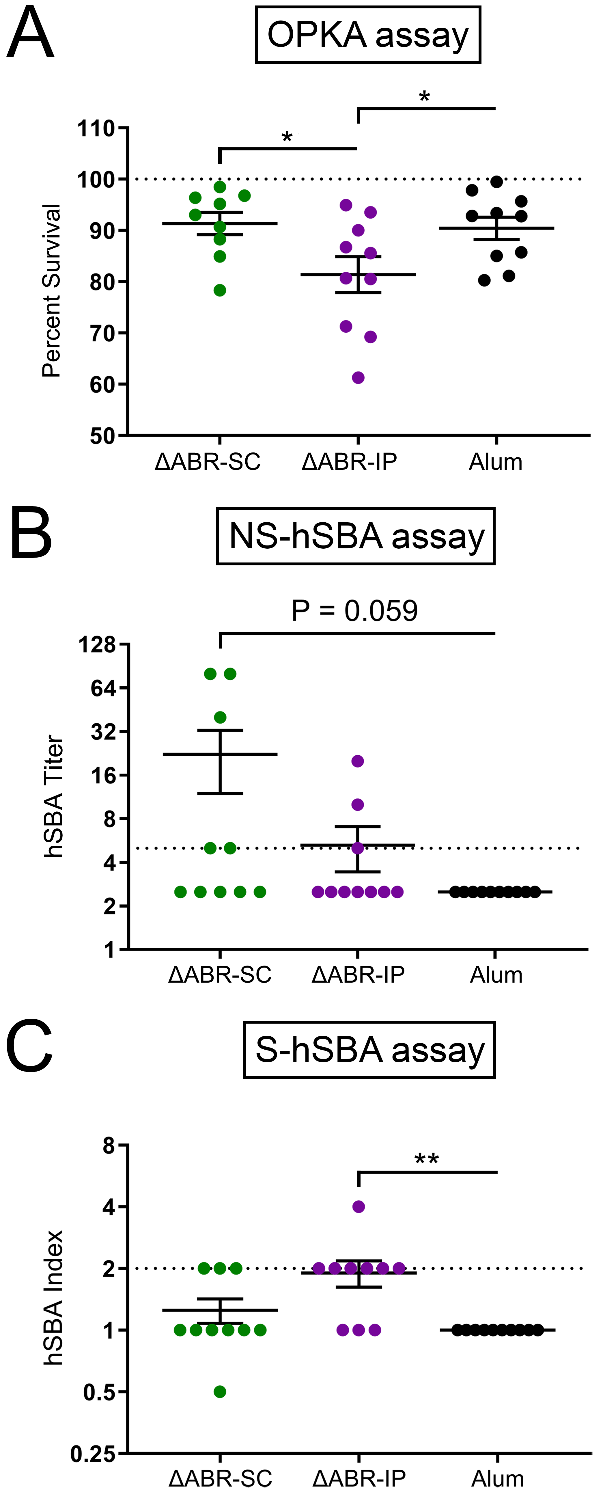


**Supplementary Figure 2.** Vaccination with ΔABR detergent-detoxified outer membrane vesicles induces functional antibody responses. *A*, Opsonophagocytic killing activity (OPKA) of mouse serum antibodies 3-weeks post-3^rd^ immunization. *P˂0.05 by 1-way ANOVA with Holm-Šidák test. *B-C*, Serum bactericidal activity (SBA) of antibodies as measured against F62 with (*B*) nonsialylated and (*C*) sialylated lipooligosaccharide. Samples with results less than the limit of detection (LOD) were imputed to a value equivalent to half the LOD. **P˂0.01 by 1-way ANOVA with Tukey’s multiple comparison test. Hashed lines for OPKA and SBA analyses represent no killing and the lower threshold of killing, respectively. Alum group represents the combined results of control mice immunized subcutaneously and intraperitoneally.


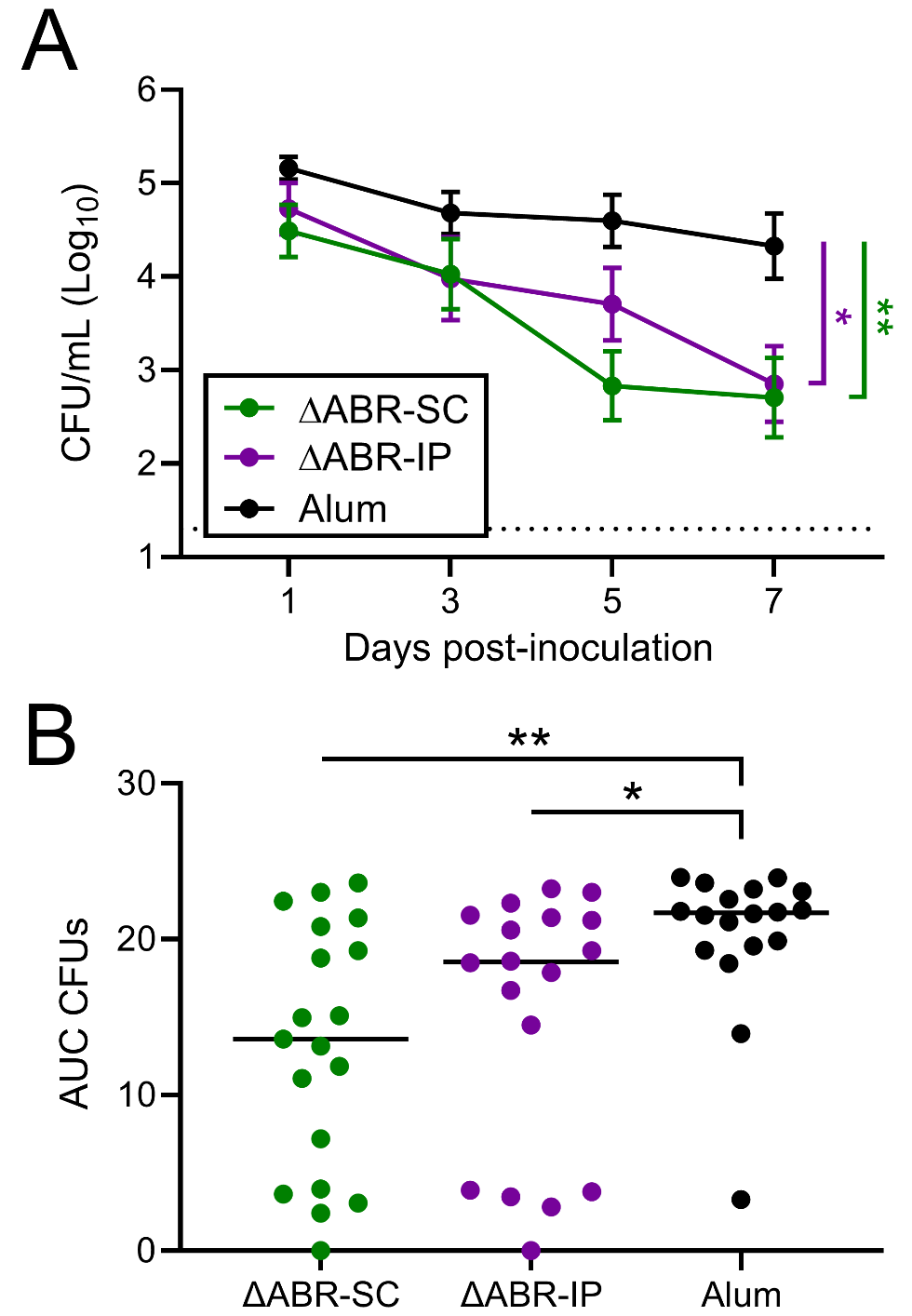


**Supplementary Figure 3.** Vaccination reduces vaginal bioburden of mice. *A*, Mean CFU/mL recovered from the lower reproductive tract +1, +3, +5, and +7 days post-challenge. Hashed line represents the lower technical limit of CFU quantification. *P˂0.05 and **P˂0.01 by 2-way repeated measures ANOVA with Bonferroni correction. *B*, AUC analysis of vaginal bioburden throughout the infection period. *P˂0.05 and **P˂0.01 by Kruskal-Wallis with Dunn’s multiple comparison test.


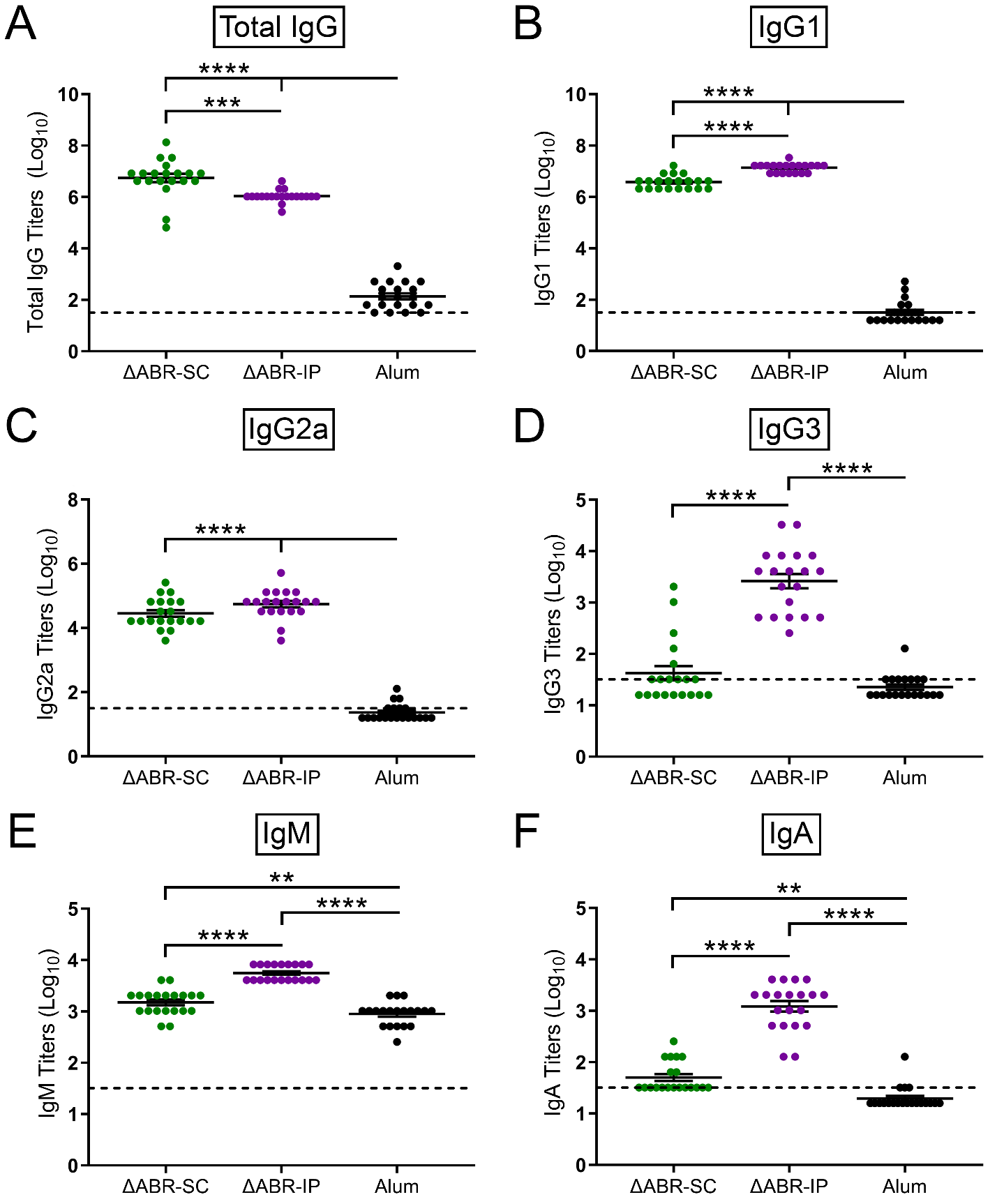


**Supplementary Figure 4.** F62-specific serum antibody levels present in mice +7 days post-bacterial challenge. **P˂0.01, ***P˂0.001, and ****P˂0.0001 by 1-way ANOVA with Tukey’s multiple comparison test. Hashed lines indicate the assay limit of detection (LOD). Samples with nonmeasurable titers were imputed to a value equivalent to half the LOD.





**Supplementary Figure 5.** Membrane preparations of five diverse gonococcal strains as probed with serum IgG and IgA antibodies. Preparations of the parental *N. meningitidis* strain MC58 were also run as a positive control. Blots probed with IgG antibodies from the ΔABR-SC and ΔABR-IP groups diluted 1:1000 recognized the same ~36–43 kDa porin antigens identified with IgA antibodies (data not shown). Arrow represents the PilQ antigen.


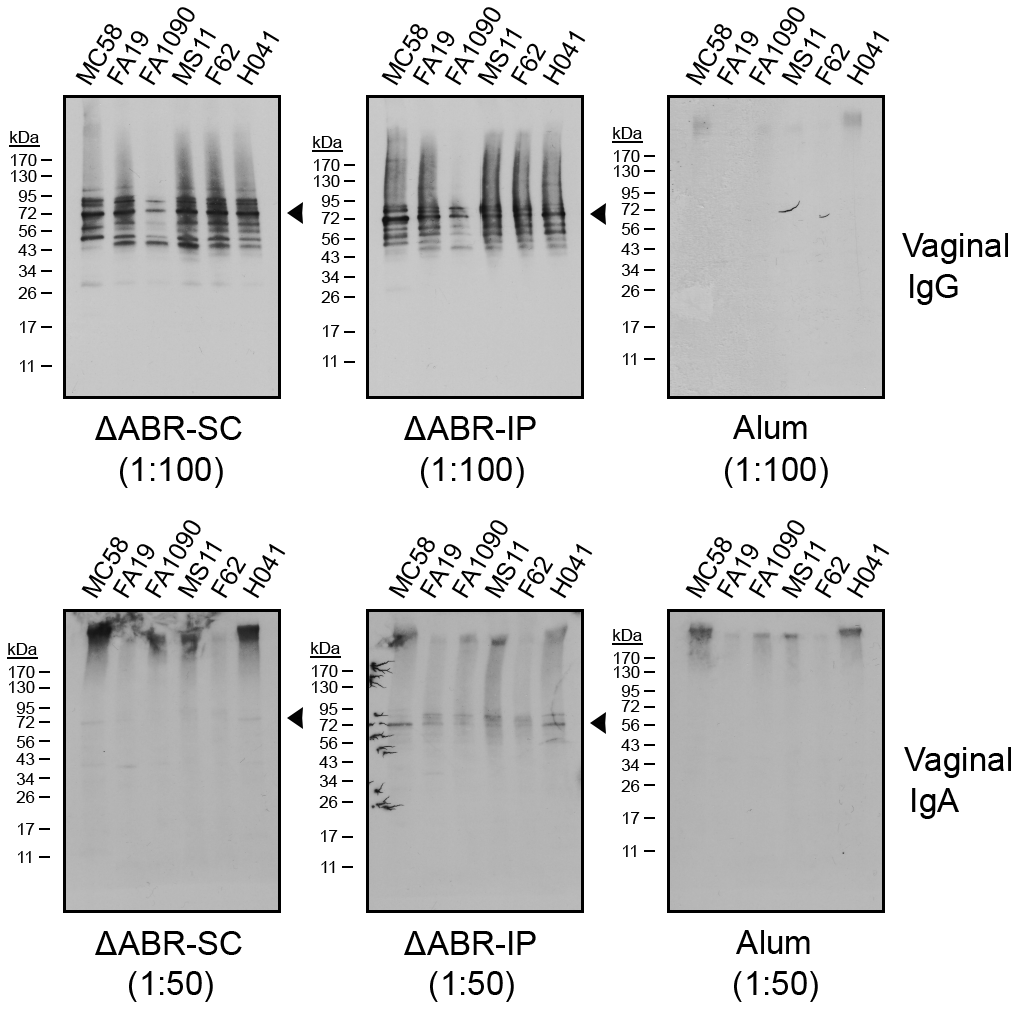


**Supplementary Figure 6.** Membrane preparations of five diverse gonococcal strains as probed with vaginal IgG and IgA antibodies. Preparations of the parental *N. meningitidis* strain MC58 were also run as a positive control. Arrow represents the PilQ antigen.


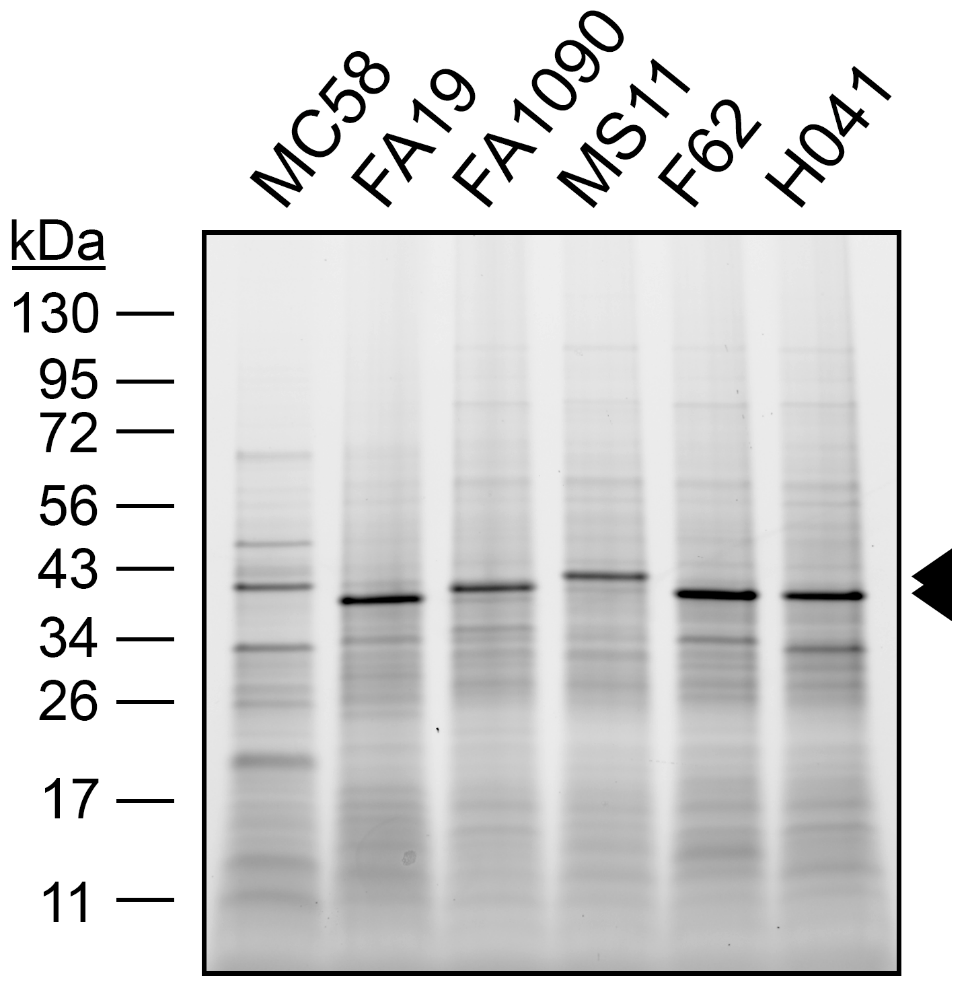


**Supplementary Figure 7.** SDS-PAGE of membrane preparations of five diverse gonococcal strains and the parental *N. meningitidis* strain MC58. Arrows represent PorB antigens of varying molecular weight.
